# P-645. Post-pandemic changes in the epidemiology of invasive pneumococcal disease in adults in Toronto, Canada

**DOI:** 10.1093/ofid/ofae631.842

**Published:** 2025-01-29

**Authors:** Altynay Shigayeva, Christopher Kandel, Shiva Barati, Gloria Crowl, Lubna Farooqi, Alyssa Golden, Kazi Hassan, Maxime Lefebvre, Angel Li, Reena Lovinsky, Nadia Malik, Irene Martin, Matthew Muller, Krystyna Ostrowska, Mare Pejkovska, Jeff Powis, David Richardson, Daniel Ricciuto, Asfia Sultana, Christie Vermeiren, Tamara Vikulova, Zoe Zhong, Allison McGeer

**Affiliations:** Sinai health, Toronto, Ontario, Canada; Michael Garron Hospital, Toronto, Ontario, Canada; Sinai Health, Toronto, Ontario, Canada; Michael Garron Hospital, Toronto, Ontario, Canada; Sinai Health, Toronto, Ontario, Canada; University of Manitoba, Winnipeg, Manitoba, Canada; Sinai Health System, Toronto, Ontario, Canada; Sinai Health, Toronto, Ontario, Canada; Sinai Health System, Toronto, Ontario, Canada; Scarborough Hospital Network, Toronto, Ontario, Canada; Sinai Health, Toronto, Ontario, Canada; National Microbiology Laboratory (NML), Winnipeg, MB, Canada; Unity Health, University of Toronto, Toronto, Ontario, Canada; Trillium Health Partners, Toronto, Ontario, Canada; Sinai Health System, Toronto, Ontario, Canada; University of Toronto, Toronto, ON, Canada; William Osler Health System, Brampton, Ontario, Canada; Lakeridge Health, Toronto, Ontario, Canada; Sinai Health, Toronto, Ontario, Canada; Shared Health Laboratories, Toronto, Ontario, Canada; Sinai Health, Toronto, Ontario, Canada; Sinai Health System, University of Toronto, Toronto, Ontario, Canada; Mt. Sinai Hospital, Toronto, Ontario, Canada

## Abstract

**Background:**

Substantial reductions in invasive pneumococcal disease (IPD) occurred during the COVID-19 pandemic. Disease incidence then increased in 2022. We assessed whether the epidemiology of IPD had changed in 2022/3 when compared to that prior to the pandemic.

**Figure 1:**
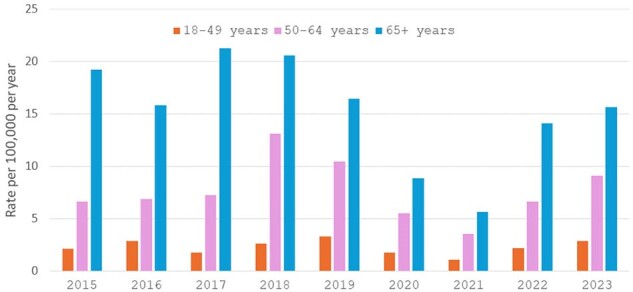
Incidence of IPD in adults by age group, Toronto and Peel region, Canada, 2015-2023

**Methods:**

TIBDN performs population-based surveillance for IPD in Toronto/Peel region (pop 4.5M). Microbiology laboratories serving area residents report sterile site isolates of *S. pneumoniae*; annual audits ensure completeness. Isolates are serotyped at Canada's National Microbiology Laboratory. Population data estimates are from Statistics Canada. We compared IPD in adults occurring in 2017-2019 to that in 2022-23.

**Figure 2:**
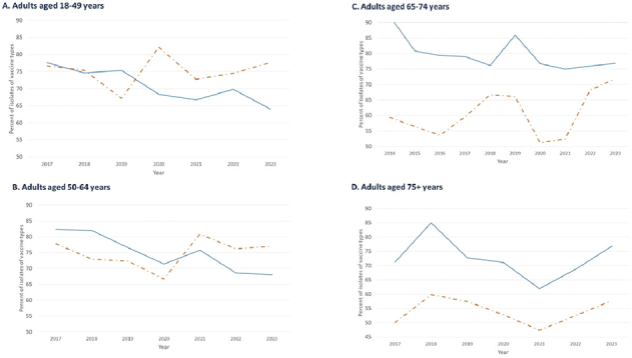
Proportion of infecting strains of serotypes included in PCV20 or PCV21/V116, by age group, 2016-2023, TIBDN Each panel shows, for a different age group, the change over time of the proportion of IPD caused by isolates of serotypes included in PCV20 (orange dashed line), and PCV21(V116) (the solid blue line).

**Results:**

Overall, 1047 IPD cases occurred in 2017-19 and 613 in 2022-23. Serotype is available for 970 (93%) and 562 (92%) of cases, and clinical information for 993 (95%) and 537 (88%) cases, respectively. Annual IPD incidence in all age groups declined during the pandemic, but has returned to 2019 rates (Figure 1). In 2022-23 compared to 2017-19, the median age of IPD patients did not differ [64y (IQR 51-75) vs 65y (52-76), P=.39] but males were more commonly affected (62% v 56%, P=.03) as were patients with cardiac (22% v 17%, P=.01) and renal (12% v 7.3% P=.007) disease (Table). Long term care (LTC) residents were less likely to have IPD in 2022-23 (1.3% v 4.5%, P< .0001). Outcomes (hospital admission, ICU admission, 30 day mortality) did not differ (Table). Between 2017-2019 and 2022-23, the proportion of IPD isolates of serotypes (STs) in PCV7 increased from 10.8% (163/970) to 18% (103/562), P< .001, while proportion of STs in PCV13 declined from 21% (207) to 16% (90), P=.01, resulting in no change in the proportion of STs in PCV20 (67% to 69%, P=.31), but a decline in the proportion in PCV21(V116) (79% to 71%, P< .001). The decline in PCV21(V116) ST proportions was more prominent in younger adults (Figure 2).

**Table.**
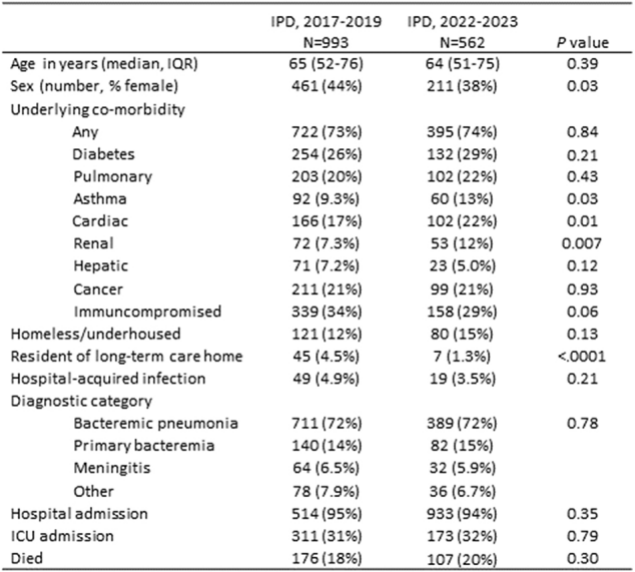
Characteristics of adults with IPD pre- (2017-2019) and post- (2022-2023) pandemic, Toronto and Peel Region, Canada

**Conclusion:**

The incidence of IPD in adults has returned to pre-pandemic levels, but differences in epidemiology persist: some (reduced disease in women and LTC residents) may be due to residual changes associated with the pandemic. Currently, PCV20 and PCV21(V116) have similar coverage in adults < 75 yrs of age in our population. Whether these changes will persist is uncertain.

**Disclosures:**

**Allison McGeer, MD**, AstraZeneca: Honoraria|GSK: Honoraria|Merck: Honoraria|Moderna: Honoraria|Novavax: Honoraria|Pfizer: Grant/Research Support|Pfizer: Honoraria|Roche: Honoraria|Seqirus: Grant/Research Support|Seqirus: Honoraria

